# Lymphocytic Choriomeningitis Virus in Employees and Mice at Multipremises Feeder-Rodent Operation, United States, 2012

**DOI:** 10.3201/eid2002.130860

**Published:** 2014-02

**Authors:** Barbara Knust, Ute Ströher, Laura Edison, César G. Albariño, Jodi Lovejoy, Emilian Armeanu, Jennifer House, Denise Cory, Clayton Horton, Kathy L. Fowler, Jessica Austin, John Poe, Kraig E. Humbaugh, Lisa Guerrero, Shelley Campbell, Aridth Gibbons, Zachary Reed, Deborah Cannon, Craig Manning, Brett Petersen, Douglas Metcalf, Bret Marsh, Stuart T. Nichol, Pierre E. Rollin

**Affiliations:** Centers for Disease Control and Prevention, Atlanta, Georgia, USA (B. Knust, U. Ströher, L. Edison, C.G. Albarino, L. Guerrero, S. Campbell, A. Gibbons, Z. Reed, D. Cannon, C. Manning, B. Petersen, S.T. Nichol, P.E. Rollin);; Georgia Department of Public Health, Atlanta (L. Edison);; Indiana Board of Animal Health, Indianapolis, Indiana, USA (J. Lovejoy, D. Metcalf, B. Marsh);; Deaconess Hospital, Evansville, Indiana, USA (E. Armeanu);; Indiana State Department of Health, Indianapolis (J. House);; Vanderburgh County Health Department, Evansville (D. Cory);; Green River District Health Department, Owensboro, Kentucky, USA (C. Horton, J. Austin);; Kentucky Department for Public Health, Frankfort, Kentucky, USA (K.L. Fowler, J. Poe, K.E. Humbaugh)

**Keywords:** Lymphocytic choriomeningitis virus, zoonotic disease, occupational health, viral meningitis, rodent-borne disease, aseptic meningitis, viruses, Indiana, United States

## Abstract

We investigated the extent of lymphocytic choriomeningitis virus (LCMV) infection in employees and rodents at 3 commercial breeding facilities. Of 97 employees tested, 31 (32%) had IgM and/or IgG to LCMV, and aseptic meningitis was diagnosed in 4 employees. Of 1,820 rodents tested in 1 facility, 382 (21%) mice (*Mus musculus*) had detectable IgG, and 13 (0.7%) were positive by reverse transcription PCR; LCMV was isolated from 8. Rats (*Rattus norvegicus*) were not found to be infected. S-segment RNA sequence was similar to strains previously isolated in North America. Contact by wild mice with colony mice was the likely source for LCMV, and shipments of infected mice among facilities spread the infection. The breeding colonies were depopulated to prevent further human infections. Future outbreaks can be prevented with monitoring and management, and employees should be made aware of LCMV risks and prevention.

## Abstract 2

Outbreaks can be prevented with strict biosecurity and microbiological monitoring.

Lymphocytic choriomeningitis virus (LCMV), a rodent-borne arenavirus, is a rare, zoonotic cause of aseptic meningitis in Europe and North America. It is carried by the common house mouse (*Mus musculus),* but other rodent species, such as hamsters and guinea pigs, can become infected and transmit infection to humans (*1*). Infected rodents shed the virus in urine, saliva, and droppings. Transplacental infection in mice results in persistently infected offspring, that shed virus throughout life (*2*). Humans become infected through close contact with infected rodents, through transplantation of infected organs, or by vertical transmission. In immunocompetent adults, infections range from mild febrile illness to aseptic meningitis; in immunosuppressed organ recipients, infections are highly fatal, and congenitally infected infants can have a range of severe birth defects (*3*).

In late April 2012, an infectious disease physician contacted the Centers for Disease Control and Prevention (CDC, Atlanta, GA, USA) about a 27-year-old woman (patient 1) who sought hospital care for fever, severe headache, photophobia, and vomiting. Cerebrospinal fluid (CSF) had elevated leukocytes (>1,000/mm^3^ [reference <5]), elevated protein (153 mg/dL [reference 12–80 mg/dL]), and negative bacterial culture. Patient 1 reported working at an Indiana rodent breeding facility (facility A). In April 2012, aseptic meningitis had been diagnosed in patient 2, who was patient 1’s domestic partner and co-worker (E. Armeanu, unpub. data). LCMV infection was suspected, and specimens were submitted to CDC for diagnostic confirmation. Serum samples from patients 1 and 2 and CSF from patient 1 were positive for LCMV IgM by ELISA, indicating recent LCMV infection. Reverse transcription PCR (RT-PCR) for LCMV was negative, indicating that viremia was no longer present.

The Vanderburgh (Indiana) County Health Department, in conjunction with the Indiana State Department of Health, Indiana Board of Animal Health, and CDC, initiated an outbreak investigation to determine the extent of LCMV infection in the staff and rodents in facility A (*4*). Trace-back investigations also identified a distributor (facility B) where live rats (*Rattus norvegicus*) and mice (*M. musculus*) from facility A were handled and packaged for sale as live and frozen animal food in 21 states (*5*). An additional mouse breeding facility in Kentucky (facility C) had shipped live mice to facility B, which redistributed them to facility A shortly before the outbreak. We describe the diagnostic and epidemiologic aspects of this investigation and the response taken to control the outbreak at these facilities.

## Materials and Methods

### Employee Serosurvey

As part of the investigation, we provided all current and former employees of facilities A, B, and C within the previous 6 months the opportunity to be interviewed and tested for LCMV as part of the investigation. County health officials administered a questionnaire that collected information about recent clinical illness and work habits at the facilities, including hygiene measures. A blood sample was collected from each interviewee and refrigerated for transport to CDC, where IgG and IgM ELISA were performed (Technical Appendix). We considered a current or former employee to be recently infected (within the past 2–3 months) with LCMV if he or she had IgM with or without IgG. An employee for whom only IgG was detectible by ELISA was considered to have had a previous infection. If employee had evidence of recent LCMV infection and had sought medical care because of illness, we reviewed his or her medical records. All employees signed an informed consent form.

### Rodent Testing

Adult breeding rodents (mice and rats) from facility A were sampled to determine LCMV infection status for each room (Technical Appendix). In accordance with the facility’s standard operating procedures for processing feeder rodents, we euthanized mice and rats with carbon dioxide gas, and the carcasses were frozen and shipped to CDC on dry ice. Carcasses were thawed, and animals were dissected under Biosafety Level 3 conditions. Heart blood and small sections of kidney and spleen were collected. Rodent specimens were tested for LCMV RNA by RT-PCR and for LCMV IgG by ELISA. RT-PCR–positive specimens were inoculated onto cell culture to isolate virus, and viral RNA was sequenced and compared with other LCMV strains (Technical Appendix).

### Statistical Analysis

All data were collected by using a computerized spreadsheet (Microsoft Excel, Microsoft, Redmond, WA, USA) and analyzed by using statistical analysis software (SAS 9.3, SAS Institute, Cary, NC, USA). Rodent location information was combined with diagnostic test results to calculate an observed prevalence of IgG seropositivity and RT-PCR positivity per room sample. Ninety-five percent CIs for the prevalence of mice with LCMV antibodies and apparent viremia per room population were estimated by using a binomial distribution equation, and estimated ranges of antibody-positive and viremic rodents per room were calculated from these ranges.

Employee questionnaire data were combined with diagnostic test results, and a case–control comparison was performed. We defined an LCMV case-patient as an employee who had detectable LCMV IgM and/or IgG and controls as employees of the same facility who had negative test results. Statistics for each facility were examined separately. Univariate analysis was conducted, and p<0.05 was considered statistically significant.

## Results

### Human LCMV Investigation

All 52 employees of facility A were tested by ELISA; Fifteen (29%) had detectable anti-LCMV antibodies; 13 had IgM and IgG, indicating recent infection. Nine IgM/IgG-positive employees reported recent clinical illness, including 5 who had sought medical treatment. Symptoms described most frequently were headache, nausea and vomiting, subjective fever, decreased appetite, diarrhea, muscle ache, and stiff neck (Table 1). Aseptic meningitis was diagnosed in 4 employees from facility A after lumbar punctures demonstrated lymphocytic CSF (Table 2). Dates of onset for laboratory-confirmed aseptic meningitis ranged from April 7 through May 14, 2012. All case-patients recovered fully.

**Table 1:** Symptoms reported by 9 employees of facility A who tested positive for LCMV IgM and reported recent illness, Indiana, USA, 2012*

Symptom or sign	Present no. (%)	Absent, no. (%)	Unknown, no.
Headache	9 (100)	0	0
Nausea/vomiting	9 (100)	0	0
Fever	8 (89)	1 (11)	0
Decreased appetite	8 (89)	1 (11)	0
Diarrhea	6 (67)	3 (33)	0
Muscle ache	6 (67)	3 (33)	0
Stiff neck	6 (67)	3 (33)	0
Joint pain	5 (56)	4 (44)	0
Malaise	5 (56)	2 (22)	2 (22)
Cough	4 (44)	5 (56)	0
Drowsiness	3 (33)	6 (67)	0
Sensory disturbance	2 (22)	6 (67)	1 (11)
Parotid pain	1 (11)	7 (78)	1 (11)
Confusion	1 (11)	8 (89)	0
Increased blood leukocyte	3 (33)	1 (11)	5 (56)
Increased CSF leukocyte	4 (44)	0	5 (56)
Increased CSF protein	4 (44)	0	5 (56)

*LCMV, lymphocytic choriomeningitis virus; CSF, cerebrospinal fluid.

**Table 2:** Demographic and clinical features of laboratory-confirmed illness in 5 facility A employees who sought health care after exposure to LCMV-infected mice, Indiana, USA, 2012*

Patient no.	Age, y/sex	Time employed	Onset date	Symptoms	Clinical findings†
1	27/F	2 y	Apr 20	Fever, muscle ache, nausea, vomiting, abdominal pain, diarrhea, malaise, headache	Lymphocytic CSF, leukocytosis (13.8), elevated protein in CSF, appendicitis, UTI
2	34/M	1.5 y	Apr 11	Fever, nausea, vomiting, malaise, headache, stiff neck	Lymphocytic CSF (1,311/µL), elevated protein in CSF
3	28/F	1.5 y	Apr 7	Fever, muscle ache, nausea, vomiting, cough, joint pain, photophobia, headache, diarrhea	UTI; no blood or CSF collected during medical evaluation
4	48/M	5 d	Apr 27	Fever, muscle ache, nausea, vomiting, abdominal pain, cough, headache, diarrhea	Lymphocytic CSF, elevated protein in CSF, mild anemia (34.9%), CT normal
5	38/M	2 mo	May 14	Fever, muscle ache, nausea, vomiting, joint pain, headache, diarrhea	Lymphocytic CSF (320/µL), elevated protein in CSF (77), leukocytosis (15.1), anemia (Hgb 11.5), CT normal

*LCMV, lymphocytic choriomeningitis virus; CSF, cerebrospinal fluid; UTI, urinary tract infection; CT, computed tomographic scan; Hgb, hemoglobin. †Lymphocytic CSF is defined as >5 lymphocytes/μL; leukocytosis is >11,000 erythrocytes/μL; and elevated protein in CSF is >60 mg/dL.

All 13 employees of facility B were tested; 1 (8%) had evidence of previous infection (LCMV IgG only). This 38-year-old woman did not recall any recent distinct clinical illness that fit the clinical profile for LCMV infection. She had not directly handled any live mice but had handled and labeled shipping packages of live mice from facilities A and C in the months before being tested.

Thirty-two of 36 facility C employees were tested; 15 (47%) had detectable LCMV antibodies, and 11 (34%) had evidence of recent LCMV infection. In facility C, 1 of the tested employees was pregnant and negative for LCMV antibodies, and a 29-year-old man who was IgG/IgM positive had visited the emergency department in May 2012 because of chest pain and headaches. Electrocardiogram, serum chemistry, and complete blood count did not show any abnormalities. Altogether, 97 employees of facilities A, B, and C were tested, and 31 (32%) had LCMV antibodies.

Job duties of employees at facilities A, B, and C ranged from administrative/managerial duties to direct handling of live and euthanized rodents to cleaning. Most employees conducted multiple duties in multiple buildings. No particular job duty was associated with LCMV infection (data not shown). Employees had worked at the facilities for 2 days–20 years, and 1 case-patient had worked at facility A for only 5 days before becoming ill. We found no association between length of time employed and previous infection, suggesting relatively recent LCMV introduction into the facilities (Table 3, Appendix).

**Table 3:** Exposures of employees at facilities A, B, and C and comparison with LCMV ELISA test results, Indiana, USA, 2012*

Variable	ELISA results†

Facility A					Facility B‡				Facility C‡			
Positive, n = 15§	Negative, n = 37	OR (95% CI)	χ^2^ p value		Positive, n = 1	Negative, n = 12	χ^2^ p value		Positive, n = 15	Negative, n = 17	OR (95% CI)	χ^2^ p value
Work													
With mice	13 (87)	28 (78)	1.86 (0.35–10.0)	0.47									
With rats	5 (33)	14 (39)	0.79 (0.22–2.79)	0.71									
Building													
1	4 (27)	8 (23)	1.22 (0.31–4.93)	0.77									
2	12 (80)	14 (40)	**6.0** **(1.43–25.19**)	**0.01**									
3	9 (60)	21 (60)	1.0 (0.29–3.44)	1.0									
4	9 (60)	20 (57)	1.12 (0.33–3.85)	0.85									
Personal protection													
Wash hands	15 (100)	37 (100)	ND	ND		1 (100)	12 (100)	ND		15 (100)	17 (100)	ND	ND
Wear gloves	15 (100)	37 (100)	ND	ND		1 (100	12 (100)	ND		14 (93)	15 (88)	1.87 (0.15–22.9)	0.62
Wear mask	10 (77)	23 (72)	1.30 (0.29–5.86)	0.73		1 (100)	9 (75)	0.9¶		6 (40)	12 (71)	0.28 (0.06–1.21)	0.08
Smoke	11 (73)	15 (42)	**3.85** **(1.02–14.45**)	**0.04**		1 (100)	1 (8)	0.18¶		3 (21)	5 (29)	0.65 (0.13–3.4)	0.47
Sex, M	10 (66)	32 (87)	0.31 (0.07–1.3)	0.21		0	9 (75)	0.31¶		10 (66)	10 (58)	1.4 (0.32–6.1)	0.21
Mean time employed, y	0.82	0.84		0.96		6	7.17	0.87		1.86	3.89		0.07
Mean age, y	33.93	36.7		0.46		38	30.75	0.54		32.33	36.76		0.18

*LCMV, lymphocytic choriomeningitis virus; OR, odds ratio. ND, not determined. Boldface indicates significant values (p<0.05). †All values are no. (%) employees who responded to the questionnaire unless otherwise indicated. ‡Housed only mice. Employees were not asked about exposures to different facility buildings, so these factors were not evaluated (blank cells). §Patients were ELISA-positive if a serum sample had detectable LCMV IgG or IgM. ¶Because of low numbers, a Fisher exact test was used to calculate p values, except for mean time employed and mean age, for which a *t* test was used.

For facility A, working in building 2 and smoking were independently associated with having recent or previous LCMV infection (Table 3, Appendix). For facilities B and C, no specific factors evaluated were associated with LCMV infection in employees tested (Table 3, Appendix). At the time of the investigation, all 97 employees reported washing hands after handling the rodents, and 90%–100% of employees reported wearing masks and gloves, although many admitted having begun using these items only when the LCMV outbreak was suspected.

### Rodent LCMV Investigation

#### Facility A

Facility A, located in Indiana, bred and raised mice and rats exclusively for sale as feeder animals for reptiles and birds of prey. Most rodents were euthanized and frozen on-site for sale as frozen feeder rodents; however, live rodents also were shipped. Live and frozen rodents were transported to facility B, also in Indiana, for storage, sale, and distribution. Facility A had 4 buildings that housed breeding rodents; each building was subdivided into rooms by species (Table 3, Appendix). At the time of the investigation, facility A housed ≈155,000 adult mice and ≈14,000 adult rats. In accordance with standard procedures, baited rodent traps were set outside and inside buildings at regular intervals throughout the facility; any mice that were caught were promptly euthanized. Rodent feed was stored indoors on pallets.

In May 2012, a total of 1,820 mice and rats from facility A were tested for LCMV IgG by ELISA and for LCMV RNA by RT-PCR. Of 1,421 mice tested, 296 (20.8%) had detectable IgG, and 10 (0.7%) had detectable RNA; apparent prevalence varied by room (Table 4). No RT-PCR–positive mice had detectable LCMV IgG. Only 1 mouse room tested (building 3, room 2) contained no IgG- or RT-PCR–positive mice, indicating that LCMV infection was widespread. The estimated number of viremic mice in a given room at the time of sampling varied from 0 to 472 (as estimated by the number of RT-PCR–positive mice) (95% CI 0–1,180 viremic mice per room) (Table 4). None of 399 rats tested were positive by ELISA or RT-PCR.

**Table 4:** Test results for LCMV from mice and rats in facility A, Indiana, USA, 2012*

Building/room	Species	No. adult rodents in room	Sample size	IgG positive			RT-PCR positive	
				No. (%)	Estimated no. per room (95% CI)†		No. (%)‡	Estimated no. per room (95% CI)†
1								
1	Rat	1,512	110	0	0 (0–41)		0	0 (0–41)
2	Rat	2,058	83	0	0 (0–72)		0	0 (0–72)
2								
1	Mouse	13,104	110	28 (25)	3,342 (2,306–4,534)		4 (4)	472 (131–1,179)
2	Mouse	10,368	110	25 (23)	2,354 (1,586–3,287)		2 (2)	187 (21–664)
3	Mouse	13,104	102	36 (35)	4,626 (3,420–5,949)		0	0 (0–380)
4	Mouse	10,368	110	42 (38)	3,961 (3,017–4,966)		1 (1)	93 (2–508)
3								
1	Mouse	14,400	110	3 (3)	389 (86–1,123)		1 (1)	130 (3–706)
2	Mouse	14,400	110	0	0 (0–389)		0	0 (0–389)
3	Mouse	14,400	110	19 (17)	2,491 (1,541–3,701)		0	0 (0–389)
4	Mouse	5,760	109	25 (23)	1,319 (887–1,843)		0	0 (0–156)
5	Mouse	14,400	110	20 (18)	2,621 (1,656–3,845)		0	0 (0–389)
6	Mouse	12,384	110	36 (33)	4,050 (2,984–5,238)		0	0 (0–334)
7	Mouse	4,320	110	1 (1)	39 (1–212)		0	0 (0–117)
4								
1	Mouse	11,520	98	37 (38)	4,355 (3,248–5,541)		1 (1)	115 (2–645)
2	Mouse	17,280	108	24 (22)	3,836 (2,557–5,391)		1 (1)	155 (3–881)
3	Rat	5,460	110	0	0 (0–147)		0	0 (0–147)
4	Rat	4,704	110	0	0 (0–127)		0	0 (0–127)

*LCMV, lymphocytic choriomeningitis virus; RT-PCR, reverse transcription PCR. †Binomial estimators were used to calculate 95% CIs for the entire adult population for each room, based on the number of test-positive animals detected with the samples obtained. ‡All RT-PCR–positive animals were LCMV IgG-negative as measured by ELISA.

LCMV was successfully isolated from 8 of 10 RT-PCR–positive mice. The sequence analysis of a 630-bp amplicon from the S-segment from the 8 isolates showed a high degree of similarity (98.6%–99.7% identity; data not shown), suggesting a single origin of LCMV. The complete S segment sequence of the prototype outbreak strain (201202467 Indiana, GenBank accession no. KF732824) was aligned and compared with 34 sequences available in GenBank, corresponding to representative strains of LCMV. The phylogenetic analysis (Figure) shows that strain 201202467 belongs to lineage I, a heavily populated LCMV clade with numerous isolates from Europe and North America (*6*).

**Figure:**
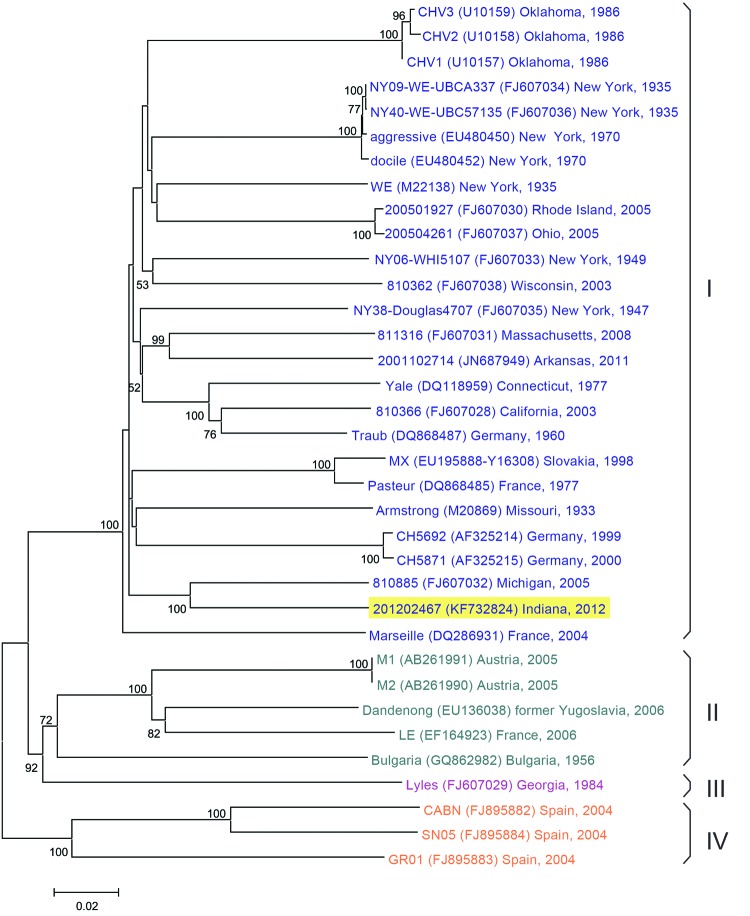
Phylogenetic tree comparing S RNA genomes of most representative lymphocytic choriomeningitis virus (LCMV) strains. Evolutionary analysis was conducted in MEGA5 Technical Appendix) by using the neighbor-joining method. Bootstrap values listed at the nodes provide statistical support for 1,000 replicates. Branches corresponding to partitions reproduced in <50% bootstrap replicates are collapsed. Scale bar indicates substitutions per site. The main LCMV lineages are indicated with roman numerals (I–IV). After the strain denomination, the location and date it was isolated are noted

After evidence of LCMV infection was detected, the Indiana Board of Animal Health (Indianapolis, IN, USA) issued quarantines and stop-movement orders on all live and frozen rodents from facility A. After animal testing was done, regulatory restrictions were lifted on rats but were retained on mice. More than 400,000 mice were euthanized and buried on site. All rodent feed and bedding was incinerated, and the facility was disinfected by using a 0.1% bleach solution. Employees were provided N95 respirators, gloves, boots, and water-resistant coveralls to wear when handling the mice and possibly contaminated equipment, and they were instructed about how to properly use the equipment and disinfect appropriately to limit their risk for infection. Once these measures were fully implemented, no additional employees became ill throughout the depopulation process, which ended on July 11, 2012.

All frozen rodents from facility A that were remaining in storage at facility B were destroyed. Live rodents originating from facility A were sold to >500 different purchasers in 21 different states (*5*).

#### Facility C

Facility C exclusively produced frozen feeder mice that were euthanized on site in Kentucky and then transported to facility B for storage and distribution. In March 2012, ≈90,000 live mice were shipped from facility C to facility A via facility B to replace breeding stock. Other than this instance, live shipments were not common. Management was similar to that of facility A. After the LCMV outbreak among facility A employees, the owners of facility C had 50 mice tested at a commercial laboratory (IDEXX RADIL, Columbia, MO, USA); 33 (66%) were antibody-positive by immunofluorescent antibody testing. No mice were tested by CDC. The owners of facility C reported this finding to state public health authorities and voluntarily depopulated all mice. Approximately 380,000 mice from facility C were euthanized, and 810,000 frozen mice stored at facility B were destroyed. Bedding was buried, and the facility was disinfected. The owners of facility C reported that 6 months before the outbreak, wild mice had infested the feed storage areas, and the owners noted occasional litters of mice born with black eyes (colony mice are albino), indicating that wild mice had interbred with the colony mice.

## Discussion

LCMV is endemic in wild *M. musculus* populations across the United States and throughout the world. Sporadic human LCMV cases occur after contact with infected wild house mice, but the virus has the potential to cause large epidemics when it enters high-density rodent populations, as in the outbreak of this report. Previous outbreaks of human disease have been linked to contact with pet hamsters and laboratory animals (*7*–*11*). In the current outbreak investigation, we found that nearly one third of rodent facility employees tested had LCMV antibodies. This overall attack rate is consistent with previous outbreaks of LCMV in hamsters and nude mice at research facilities (*10*,*12*,*13*).

Employees of rodent breeding facilities of all kinds should be aware of the risks posed by exposure to rodents infected with LCMV, and monitoring programs should be in place to detect and control infections in rodents. Commercial laboratory rodent breeding colonies have developed management practices to avoid contact between wild mice and colony animals; the US Department of Health and Human Services and the Federation of European Laboratory Animal Science Associations recommend routine virologic and serologic monitoring to detect pathogens, including LCMV (*2*,*14*). Facilities producing rodents for the pet and feeder-rodent industries should adapt these practices to avoid such outbreaks. When LCMV or LCMV antibodies are detected in rodents or employees of a rodent breeding operation, all animal-handling personnel should wear protective equipment, including a respirator. After personal protective gear and training in its proper use were provided to facility employees, LCMV infection was not laboratory confirmed in any additional employees of facility A throughout the depopulation process, which implies that use of such equipment can reduce the risk for infection.

In facility A, many mice had LCMV antibodies, and several were viremic at the time of the investigation. The rodent testing results fit with the human testing results; that is, working in building 2, which had the highest prevalence of LCMV antibody-positive and RT-PCR–positive mice, was significantly associated with employee infections. Of the ≈13,000 adult mice in room 1 of building 2, ≈131–1,179 were viremic. In such a high-density environment, the virus can be present in aerosol form (*15*), which explains why many employees of facilities A and C had detectable antibodies. In facility B, which only transiently held live mice for further distribution, only 1 (8%) of 13 employees had detectable LCMV antibodies, suggesting that less intensive exposure to mice put employees at lower risk for infection. Smoking, a risk factor for many bacterial and viral infections, including tuberculosis, pneumococcal pneumonia, and influenza (*16*), was associated with LCMV infection in facility A employees. The structural changes to the respiratory epithelium and modulation of immune function hypothetically impair the smoker’s immune response. The physical act of smoking also might facilitate transfer of pathogens from the hands to the mouth.

The proportion of infected employees in whom clinical illness developed varied by facility. In facility A, 69% of employees who had recent infection reported illness, and 33% had symptoms severe enough to cause them to seek medical care. Conversely, all facility C employees appeared to have asymptomatic infections, except for 1 with nonspecific illness. Previous LCMV outbreak investigations have found various rates of clinical illness (*9*,*10*,*13*), with asymptomatic infections of 25%–55%. Because employee age and sex did not differ among facilities, the reason for the difference in disease manifestation remains unclear. The longevity of LCMV IgM detectable in peripheral blood is not well-established for humans; thus, some employees with IgM who did not develop symptoms might have been infected previously. 

In immunocompetent adults, the neurologic form of LCMV infection classically has biphasic features consisting of a nonspecific initial phase, with fever, myalgia, and headache most commonly observed, and can include nausea and/or vomiting and retroorbital pain (*8*,*17*). Symptoms may subside after several days to be followed by a neurologic phase comprising meningeal symptoms with fever, headache, nuchal rigidity, vomiting, and light sensitivity. In LCMV patients in whom aseptic meningitis is diagnosed, CSF characteristically shows a lymphocytic pleocytosis, and elevated protein and decreased glucose also might be present (*1*,*3*); in this report, all 4 case-patients on whom lumbar punctures were performed had lymphocytic CSF with elevated protein. Symptoms frequently reported by case-patients in this outbreak included headache; fever; and abdominal symptoms, such as nausea, vomiting, and diarrhea. Such nonspecific symptoms obscure the clinical diagnosis. A thorough clinical history covering relevant animal contacts remains vital to determining the source and appropriate etiologies to test for in work-ups of aseptic meningitis.

Although all case-patients in this LCMV outbreak recovered from their illness, the specter of more severe disease manifestations remains a cause for concern. Infections during pregnancy can result in spontaneous abortion or characteristic congenital defects, such as chorioretinitis, microencephaly or macroencephaly, and hydrocephalus (*17*). Mental retardation, vision deficits, cerebral palsy, and epilepsy are potential lifelong manifestations (*18*). Since 2005, five outbreaks of LCMV have occurred after organ transplantation, resulting in the death of 14 (82%) of 17 organ recipients (*19*–*22*). Concern about these severe forms of LCMV infection led the outbreak response team to recommend depopulating the mouse breeding facilities and to conduct a trace-forward investigation of live mice that were sold and shipped from facility B (*6*).

LCMV is transmitted horizontally and vertically in affected rodents (*16*,*23*). Horizontal infections, acquired through direct contact with infected rodents or indirect contact with contaminated fomites, can lead rodents to shed infectious virus for a few weeks to a few months. When mice are exposed in utero to LCMV, they become persistently infected and shed the virus throughout their lives, including to all offspring, which also will be persistently infected (*2*,*24*,*25*). Infections in rodents are inapparent. As a result, when LCMV is introduced to a high-density environment, such as the breeding colonies of this outbreak, the number of infected rodents can silently reach very high numbers and thus pose a risk to the humans coming in contact with them.

The primary goal of the rodent sampling scheme was to detect LCMV infection in the colony; only adult mice were sampled because we assumed that they would have the greatest likelihood of having detectable antibodies. If younger mice had been tested, the seroprevalence is likely to have been lower and the proportion of viremic animals higher. In this investigation, 21% of adult mice tested had antibodies; this prevalence is higher than that found in wild mouse populations not associated with human infections (2%–9%) (*26*–*28*).

These molecular investigations demonstrated a unique strain of LCMV, 201202467, in facility A mice, which suggests a single introduction and transmission event throughout the breeding facility. Although we were unable to sample mice from facility C, it is likely that the outbreak strain was the same as in facility A and was introduced during the wild mouse infestation that had occurred. Molecular analysis also shows the close relationship of strain 201202467 with another LCMV strain isolated from a mouse found in an infested house in Michigan in 2005 (*29*). The geographic distribution of this LCMV strain throughout North America is not known (*7*).

As in previous outbreaks, interactions between wild mice and colony rodents frequently introduce LCMV into breeding colonies (*16*). In the absence of control measures and monitoring, movements of live mice among breeder mice can contribute to the spread of potentially infected mice. No human or rodent LCMV vaccine is available, and no treatment exists. Therefore, prevention measures are necessary and must rely on wild rodent exclusion, infection control, and microbiological monitoring (*2*). When LCMV antibodies are detected in colony mice, transmission must be assumed to be ongoing, and all possibly infected or exposed rodents should be removed from the colony by euthanasia and disposal to mitigate human risk (*23*,*30*).

In conclusion, laboratory and epidemiologic investigations effectively identified a large outbreak of LCMV in 3 commercial mouse breeding facilities and associated infections in several employees. The presumptive source of virus introduction was contact between wild mice and colony mice, and the outbreak spread among facilities when mice were transported for use as breeding stock. The breeding colonies were depopulated to prevent further human infections. Future outbreaks can be prevented with strict biosecurity and microbiological monitoring, and employees should be made aware of the symptoms of LCMV infection and prevention measures.

Technical Appendix Detailed animal sampling and laboratory methods.

## References

[1] Fisher-Hoch SP. Arenavirus pathophysiology. In: Salvato MS, editor. The Arenaviridae. New York: Plenum Press; 1993. p. 308–10.

[2] Shek WR. Lymphocytic choriomeningitis virus. In: Waggie K, Kagiyama N, Allen AM, Nomura T, editors. Manual of microbiologic monitoring of laboratory animals. 2nd ed. Bethesda (MD): US Department of Health and Human Services; 1994. p. 35–42.

[3] Bonthius DJ. Lymphocytic choriomeningitis virus: a prenatal and postnatal threat. Adv Pediatr. 2009;56:75–86 . 10.1016/j.yapd.2009.08.007 19968943

[4] Centers for Disease Control and Prevention. Notes from the field: lymphocytic choriomeningitis virus infections in employees of a rodent breeding facility—Indiana, May–June 2012. MMWR Morb Mortal Wkly Rep. 2012;61:622–3 . 22895387

[5] Edison L, Knust B, Petersen B, Gabel J, Manning C, Drenzek C, Trace-forward investigation of mice in response to lymphocytic choriomeningitis virus outbreak. Emerg Infect Dis. 2014;20:291–5. 10.3201/eid2002.130861 24447898 PMC3901476

[6] Albariño CG, Palacios G, Khristova ML, Erickson BR, Carroll SA, Comer JA, High diversity and ancient common ancestry of lymphocytic choriomeningitis virus. Emerg Infect Dis. 2010;16:1093–100. 10.3201/eid1607.091902 20587180 PMC3321910

[7] Amman BR, Pavlin BI, Albariño CG, Comer JA, Erickson BR, Oliver JB, Pet rodents and fatal lymphocytic choriomeningitis in transplant patients. Emerg Infect Dis. 2007;13:719–25 and. 10.3201/eid1305.061269 17553250 PMC2738461

[8] Biggar RJ, Woodall JP, Walter PD, Haughie GE. Lymphocytic choriomeningitis outbreak associated with pet hamsters. Fifty-seven cases from New York State. JAMA. 1975;232:494–500 and. 10.1001/jama.1975.03250050016009 1173141

[9] Deibel R, Woodall JP, Decher WJ, Schryver GD. Lymphocytic choriomeningitis virus in man. Serologic evidence of association with pet hamsters. JAMA. 1975;232:501–4 and. 10.1001/jama.1975.03250050023010 1091750

[10] Dykewicz CA, Dato VM, Fisher-Hoch SP, Howarth MV, Perez-Oronoz GI, Ostroff SM, Lymphocytic choriomeningitis outbreak associated with nude mice in a research institute. JAMA. 1992;267:1349–53 and. 10.1001/jama.1992.03480100055030 1740856

[11] Gregg MB. Recent outbreaks of lymphocytic choriomeningitis in the United States of America. Bull World Health Organ. 1975;52:549–53 . 1085210 PMC2366644

[12] Vanzee BE, Douglas RG, Betts RF, Bauman AW, Fraser DW, Hinman AR. Lymphocytic choriomeningitis in university hospital personnel. Clinical features. Am J Med. 1975;58:803–9 and. 10.1016/0002-9343(75)90635-X 1138538

[13] Hinman AR, Fraser DW, Douglas RG, Bowen GS, Kraus AL, Winkler WG, Outbreak of lymphocytic choriomeningitis virus infections in medical center personnel. Am J Epidemiol. 1975;101:103–10 . 1092154 10.1093/oxfordjournals.aje.a112076

[14] Nicklas W, Baneux P, Boot R, Decelle T, Deeny AA, Fumanelli M, Recommendations for the health monitoring of rodent and rabbit colonies in breeding and experimental units. Lab Anim. 2002;36:20–42 and. 10.1258/0023677021911740 11831737

[15] Bowen GS, Calisher CH, Winkler WG, Kraus AL, Fowler EH, Garman RH, Laboratory studies of a lymphocytic choriomeningitis virus outbreak in man and laboratory animals. Am J Epidemiol. 1975;102:233–40 . 1163529 10.1093/oxfordjournals.aje.a112152

[16] Arcavi L, Benowitz NL. Cigarette smoking and infection. Arch Intern Med. 2004;164:2206–16 and. 10.1001/archinte.164.20.2206 15534156

[17] Barton LL, Mets MB. Congenital lymphocytic choriomeningitis virus infection: decade of rediscovery. Clin Infect Dis. 2001;33:370–4 and. 11438904 10.1086/321897

[18] Bonthius DJ, Wright R, Tseng B, Barton L, Marco E, Karacay B, Congenital lymphocytic choriomeningitis virus infection: spectrum of disease. Ann Neurol. 2007;62:347–55 and. 10.1002/ana.21161 17557350

[19] Fischer SA, Graham MB, Kuehnert MJ, Kotton CN, Srinivasan A, Marty FM, Transmission of lymphocytic choriomeningitis virus by organ transplantation. N Engl J Med. 2006;354:2235–49 and. 10.1056/NEJMoa053240 16723615

[20] Palacios G, Druce J, Du L, Tran T, Birch C, Briese T. A new arenavirus in a cluster of fatal transplant-associated diseases. N Engl J Med. 2008;358:991–8 and. 10.1056/NEJMoa073785 18256387

[21] Centers for Disease Control and Prevention. Brief report: lymphocytic choriomeningitis virus transmitted through solid organ transplantation—Massachusetts, 2008. MMWR Morb Mortal Wkly Rep. 2008;57:799–801 . 18650788

[22] Macneil A, Stroeher U, Farnon E, Campbell S, Cannon D, Paddock C, Solid organ transplant–associated lymphocytic choriomeningitis, United States, 2011. Emerg Infect Dis. 2012;18:1256–62 and. 10.3201/eid1808.120212 22839997 PMC3414043

[23] Institute for Laboratory Animal Research. Health surveillance programs. In: Infectious diseases of mice and rats. Washington (DC): National Academies Press; 1991. p. 21–30.

[24] Skinner HH. Monitoring mouse stocks for lymphocytic choriomeningitis virus—a human pathogen. Lab Anim. 1971;5:73–87 and. 10.1258/002367771781006618 5001340

[25] Kang SS, McGavern DB. Lymphocytic choriomeningitis infection of the central nervous system. Front Biosci. 2008;13:4529–43 and. 10.2741/3021 18508527 PMC5279998

[26] Childs JE, Glass GE, Korch GW, Ksiazek TG, Leduc JW. Lymphocytic choriomeningitis virus infection and house mouse (*Mus musculus*) distribution in urban Baltimore. Am J Trop Med Hyg. 1992;47:27–34 . 1636880 10.4269/ajtmh.1992.47.27

[27] Becker SD, Bennett M, Stewart JP, Hurst JL. Serological survey of virus infection among wild house mice (*Mus domesticus*) in the UK. Lab Anim. 2007;41:229–38 and. 10.1258/002367707780378203 17430622

[28] Morita C, Tsuchiya K, Ueno H, Muramatsu Y, Kojimahara A, Suzuki H, Seroepidemiological survey of lymphocytic choriomeningitis virus in wild house mice in China with particular reference to their subspecies. Microbiol Immunol. 1996;40:313–5 and. 10.1111/j.1348-0421.1996.tb03342.x 8709868

[29] Foster ES, Signs KA, Marks DR, Kapoor H, Casey M, Stobierski MG, Lymphocytic choriomeningitis in Michigan. Emerg Infect Dis. 2006;12:851–3 and. 10.3201/eid1205.050794 16704853 PMC3374428

[30] Centers for Disease Control and Prevention. Update: interim guidance for minimizing risk for human lymphocytic choriomeningitis virus infection associated with pet rodents. MMWR Morb Mortal Wkly Rep. 2005;54:799–801 . 16107785

