# Supplementary material for: Lymphocytic Choriomeningitis Virus in Employees and Mice at Multipremises Feeder-Rodent Operation, United States, 2012
Source: Emerg Infect Dis. 2014 Feb;20(2):240–7. doi: 10.3201/eid2002.130860 (PMC3901486; doi:10.3201/eid2002.130860)
Supplement: Technical Appendix — Detailed animal sampling and laboratory methods. [file 13-0860-Techapp-s1.pdf]

Article DOI: http://dx.doi.org/10.3201/eid2002.130860

Lymphocytic Choriomeningitis Virus in Employees and Mice at Multipremises Feeder-Rodent Operation, USA, 2012

Technical Appendix

Detailed Animal Sampling and Laboratory Methods

Facility A Rodent Sampling

In facility A, numbers of adult breeding rodents in each room were estimated. Each room was treated as a discrete population. Hypergeometric probability equations were used to determine sample size, similar to a previous lymphocytic choreomeningitis virus (LCMV) investigation (*1*). Given the estimated range of adult rodents per room (1,500–15,000), a sample size of 110 animals was estimated to have a 96.5%–96.9% probability of detecting at least 1 antibody-positive animal if the overall seroprevalence was $\geq$3%. Systematic sampling was used to select at least 1 adult animal from each rack in the room, with varying pan locations within the rack. Juvenile animals were not sampled.

Diagnostic Assays

ELISA

Human serum was tested for LCMV IgM and IgG by using in-house ELISA as described (*2*). Mouse and rat serum was tested with a modified LCMV IgG ELISA by using goat antimouse conjugate (Cat 31446, Pierce, Rockford, IL, USA) or antirat conjugate (Cat 14-16-06, Kirkegaard & Perry Laboratories, Gaithersburg, MD, USA).

RT-PCR and Virus Genome RNA Sequencing

Tissue specimens of mouse liver and spleen (≈100 mg) were collected in 500 µL of lysis binding solution (Ambion Life Technologies, Austin, TX, USA) and homogenized in a high-throughput tissue grinder (Geno/Grinder 2000, SPEX SamplePrep, Metuchen, NJ, USA). Total RNA was extracted with MagMax-96 total RNA isolation kit (SuperScrip III One-Step RT-PCR System with Platinum Taq High Fidelity, Invitrogen Life Technologies, Grand Island, NY, USA),

following the manufacturer's instructions. A 655-bp product corresponding to the nucleoprotein gene was amplified by using a generic primer set capable of amplifying all known strains of LCMV. The primers used in these reactions were as follows: LCMV1748F (AIATIATRCARTCCATRAGIGCRCA) and LCM2377R (TCIGGIGARGGITGGCCITAYAT). The RT-PCR was done as described (*1*) with SuperScript III One-Step RT-PCR System with Platinum Taq High Fidelity (Invitrogen Life Technologies), according to the manufacturer instructions. The amplified nucleoprotein gene fragments from 8 positive samples were sequenced by using standard dideoxy-sequencing techniques (Invitrogen Life Technologies). To further characterize the LCMV strain, the complete S RNA (≈3,411 nt) from 1 representative sample (201202467) was amplified by RT-PCR, as described (*3*), by using the arenavirus standard primer Are19c (CGCACAGTGGATCCTAGGC). Multiple sequence alignments and phylogenetic analysis were done using MEGA4 (*4*).

Virus Isolation

For all mice found to have virus RNA detected by RT-PCR, 100 mg of mixed spleen and liver tissues were ground manually in Hank's Balanced Salt Solution and centrifuged. 100 µL of the resulting tissue homogenate suspension was inoculated on confluent Vero E6 cells. Flasks were incubated at 37°C and monitored for cytopathic effects. On day 7, culture medium was changed and cells checked by immunofluorescence assay (IFA) for the presence of LCMV. Negative cultures were kept until day 14 for a final IFA before being discarded. Positive cultures were harvested and stored.

References

1. Amman BR, Pavlin BI, Albariño CG, Comer JA, Erickson BR, Oliver JB, et al. Pet rodents and fatal lymphocytic choriomeningitis in transplant patients. Emerg Infect Dis. 2007;13:719–25. http://dx.doi.org/10.3201/eid1305.061269

2. Park JY, Peters CJ, Rollin PE, Ksiazek TG, Katholi CR, Waites KB, et al. Age Distribution of lymphocytic choriomeningitis virus serum Antibody in Birmingham, Alabama: evidence of a decreased risk of infection. Am J Trop Med Hyg. 1997;57:37–41.

3. Albariño CG, Palacios G, Khristova ML, Erickson BR, Carroll SA, Comer JA, et al. High diversity and ancient common ancestry of lymphocytic choriomeningitis virus. Emerg Infect Dis. 2010;16:1093–100. http://dx.doi.org/10.3201/eid1607.091902

4. Tamura K, Dudley J, Nei M, Kumar S. MEGA4: molecular evolutionary genetics analysis (MEGA) software version 4.0. Mol Biol Evol. 2007;24:1596–9. http://dx.doi.org/10.1093/molbev/msm092
